# Correlation of Sarcopenia With Modified Frailty Index as a Predictor of Outcome in Critically Ill Elderly Patients: A Cross-Sectional Study

**DOI:** 10.7759/cureus.19065

**Published:** 2021-10-26

**Authors:** Shreerang Bhurchandi, Sunil Kumar, Sachin Agrawal, Sourya Acharya, Shraddha Jain, Dhruv Talwar, Sunayana Lomte

**Affiliations:** 1 Department of Medicine, Jawaharlal Nehru Medical College, Datta Meghe Institute of Medical Sciences, Wardha, IND; 2 Department of Otorhinolaryngology, Jawaharlal Nehru Medical College, Datta Meghe Institute of Medical Sciences, Wardha, IND

**Keywords:** critically ill, elderly, modified frailty index, outcomes, sarcopenia

## Abstract

Introduction: Frailty phenotype represents weight loss, decreased functional and physical capacity and activity, falls, slow gait, and memory impairment. Sarcopenia is a generalized and progressive loss of skeletal muscle mass, strength, and function, which occurs due to primary effects of aging and secondary effects of other causes including diseases, malnutrition, and inactivity.

Materials and Methods: This prospective cross-sectional study was performed on 70 critically ill geriatric patients (of age > 60 years) admitted in Medicine ICU (MICU) from December 2020 to May 2021 at a rural medical school at Wardha in central rural India. We assessed sarcopenia in all the patients by the European Working Group on Sarcopenia in Older People (EWGSOP) criteria and compared it with the modified frailty index. All the patients were divided in sarcopenic and non-sarcopenic groups; frailty index was applied, and outcomes were measured in terms of mortality, the need for ventilation, and length of ICU stay.

Results: In this study, the mean age of the patients was 68.61 ± 5.88 years in the sarcopenic group and 69.10 ± 6.31 years in the non-sarcopenic group. Eighteen (42.86%) patients in the sarcopenic group were severely frail, whereas eight (28.57%) patients in the non-sarcopenic group (p = 0.532) were not. In the sarcopenic severely frail group, mortality was six (14.29%), and eight (19.05%) required ventilation (p = 0.001), whereas in the non-sarcopenic severely frail group, mortality as well as the need for ventilation were four (14.29%) (p = 0.0001). Total duration of ICU stays was 4.30 ± 1.07 days in the sarcopenic group (n = 42), whereas it was 3.85 ± 1.23 days in the non-sarcopenic group (n = 28) (p = 0.10).

Conclusion: Our study found that critically ill patients with sarcopenia had more tendency to become frail, thereby increased risk of mortality. A timely routine assessment for sarcopenia upon ICU admission may provide an important prognostic factor in patient outcomes.

## Introduction

Frailty phenotype represents weight loss, decreased functional and physical capacity and activity, falls, slow gait, and memory impairment [1]. Sarcopenia is a generalized and progressive loss of skeletal muscle mass, strength, and function, which occurs due to the primary effects of aging and secondary effects of other causes including diseases, malnutrition, and inactivity [2]. Aging leads to an increase in the prevalence of sarcopenia, and it is approximately about 5%-13% in the sixth and seventh decades of life [2]. Its prevalence for people aged greater than 80 years may be as high as 50% [3]. Even in critically ill patients also, its prevalence is 5%-13% [3].

To define sarcopenia, the Asian Working Group for Sarcopenia (AWGS) made a criterion which states that a person is said to be sarcopenic if he/she has lower physical performance and/or lower muscle mass plus lower muscle strength. In 2010, the European Working Group on Sarcopenia in Older People (EWGSOP) gave a worldwide accepted definition of sarcopenia, which was later altered by them (EWGSOP2) in 2018. It was re-defined as lower muscle strength, lower muscle quantity or quality, and lower physical performance [3]. But this title is especially denoted for geriatric patients; previously, it was not been very well defined in the critically ill patients of ICU [4]. Like sarcopenia, a condition termed secondary sarcopenia is been defined in ICU patients and is also named as ICU-acquired weakness (ICU-AW) [4]. As there is a scarcity of studies in this area, the informative data for primary sarcopenia or sarcopenia related to age in patients of ICU is not enough [4]. Some studies believe that frailty in the patients who are critically ill is required for evaluation and its correlation with endpoints such as existence, life quality, and its relation with the utilization of the resources like duration of mechanical ventilation, the length of ICU stay, and hospitalization [5]. The assessment of sarcopenia and its correlation with the modified frailty index in critically ill elderly patients attending the ICUs will help in correlating its importance with frailty and various other systemic components of frailty. So that earlier evaluation of sarcopenia and its management can be planned to improve the frailty.

## Materials and methods

A prospective cross-sectional study was performed on all the critically ill geriatric population (of age > 60 years) who were admitted to the Medicine ICU (MICU) of a rural medical school in central India for six months duration after getting ethical committee approval from Datta Meghe Institute of Medical Sciences (Deemed to be University)/Institutional Ethical Committee/2020-21/31[DMIMS(DU)/IEC/2020-21/31], who satisfied the various inclusion and exclusion criteria for selection. Also written informed consent was then taken from the patients participating in this study.

A detailed history in the form of age, sex, occupation, the reason for hospital visit/admission, diabetes mellitus (DM), hypertension (HTN), chronic obstructive airway disease (COAD), asthma, cardiovascular and cerebrovascular diseases, medications, etc. was recorded in proforma. We assessed frailty by modified frailty index, i.e., Frailty Index in Rural Elderly - Mental status, Activities of daily living, Depression, and Events (FIRE-MADE) [6]. We assessed sarcopenia in all the patients and divided the patients into sarcopenic and non-sarcopenic groups as per strength, assistance with walking, rising from a chair, climbing stairs, and falls (SARC-F) questionnaire recommended by the EWGSOP2 criteria. Both the groups were compared with the modified frailty index (FIRE-MADE) where a score < 0.25 corresponded to fit; 0.25-0.49 represented mild frailty; 0.5-0.69 represented moderate frailty; and >0.7 corresponded to severe frailty. The parameters of FIRE-MADE with their scoring are given in Table 1. Outcomes were measured in terms of mortality, the need for ventilation, and the length of ICU stay in all the patients.

**Table 1:** Components of the FIRE-MADE frailty index The index was calculated as the sum of the presence of the deficits, which is divided by the total number of all the potential deficits (10 in this model). Score < 0.25 corresponded to fit; 0.25-0.49 represented mild frailty; 0.5-0.69 represented moderate frailty; and >0.7 corresponded to severe frailty. DM, Diabetes mellitus; IHD, ischemic heart disease; COPD, chronic obstructive pulmonary disease; FIRE-MADE, Frailty Index in Rural Elderly – Mental status, Activities of daily living, Depression, and Events.

S. No.	Parameters	Score (0 to 10)
1	Mental status by Mini Mental State Examination (MMSE) score: 27-30 = Normal; <27 = impaired cognitive function	Normal = 0; Impaired = 1
2	Activities of daily living (ADL) score (bathing, dressing, feeding, going to the toilet, transferring, urinary incontinence)	No help = 0; Need help = 1; on any of the following parameters
3	Geriatric depression scale (GDS) (short version) score (>5 = probable depression)	No = 0, Yes = 1
4	Events	
A	Polypharmacy	No = 0, Yes = 1
B	DM	No = 0, Yes = 1
C	IHD	No = 0, Yes = 1
D	COPD/Asthma	No = 0, Yes = 1
E	Stroke	No = 0, Yes = 1
F	Cancer	No = 0, Yes = 1
G	Others	No = 0, Yes = 1

Sarcopenia was assessed by EWGSOP2 criteria, which includes (1) lower strength of muscle, (2) lower quantity or quality of muscle, and (3) lower performance physically. Sarcopenia was probably recognized by these criteria. (1) Diagnosis was validated by adding evidence of the criteria. (2.) When the criteria 1- 3 get fulfilled, then the sarcopenia was considered as severe [3]. The strength of the muscle was assessed by handgrip strength and upper & lower limb muscle strength or power [7]. Physical performance was assessed by the short physical performance battery (SPPB) that measures in the group, in which the results of balance test, chair stand, and gait speed were combined [7-10]. Hand grip strength (HGS) was assessed as per the neurological examination. Patients were asked to grip the examiner’s finger perfectly in a standing position with the forearms away from the body at the level of the thigh. Participants were then asked to apply the maximum grip strength and hold it for 3-5 seconds, and the examiner tried to free his fingers from the grip. If low grip strength was observed, a maximum of three attempts was given to the participant with at least 30 seconds of resting interval. Upper limb and lower limb muscle strength or power of major muscles were assessed by the neurological examination methods by the standard protocol for tone and power. Muscle mass or quantity was assessed by mid-arm circumference and calf circumference measurements. The use of the SARC-F questionnaire is recommended by the EWGSOP2, which was used to demonstrate self-documentations from subjects on sarcopenia characteristic signs (score ≥ 4) [7]. SARC-F questionnaire with scoring is given in Table 2 [8].

**Table 2:** SARC-F Sarcopenia questionnaire (0-10 points) SARC-F, Strength, assistance with walking, rising from a chair, climbing stairs, and falls.

Component	Question	Scoring
Strength	How much difficulty do you have in lifting and carrying 10 pounds?	None = 0; Some = 1; A lot or unable = 2
Assistance in walking	How much difficulty do you have walking across a room?	None = 0; Some = 1; A lot, use aids, or unable = 2
Rise from a chair	How much difficulty do you have transferring from a chair or bed?	None = 0; Some = 1; A lot or unable without help = 2
Climb stairs	How much difficulty do you have climbing a flight of 10 stairs?	None = 0; Some = 1; A lot or unable = 2
Falls	How many times you have fallen in the last year?	None = 0; 1-3 = 1; four or more falls = 2

Anthropometric measurements

Mid-upper Arm Circumference Measurement (MUAC)

MUAC was measured with the elbow in a relaxed position with the arm hanging freely by the side at the midpoint of halfway between the tip of the acromion process and the tip of the olecranon process perpendicular to the long axis of the upper arm, and values were recorded nearest to 0.1 cm. Mid-upper arm circumference of both the right and left sides were taken twice, and an average was recorded. Mid-upper arm circumference of less than 23 cm for males and less than 22 cm for females was considered as loss of muscle mass [11-13].

Calf Circumference (CC)

With the help of elastic measuring tape, the CC was measured at the greatest girth of the calf when the subject was standing in an upright position and the weight of the body was evenly distributed on both legs. Two measurements of both right and left sides were taken, and an average was recorded. CC of less than 35 cm for males and less than 33 cm for females was considered as a loss of muscle mass [14]. Physical performance was assessed by SPPB, which is a group of measures that combines the results of chair stand, balance test, and gait speed [7,9].

To Check the Balance

**Figure 1:**
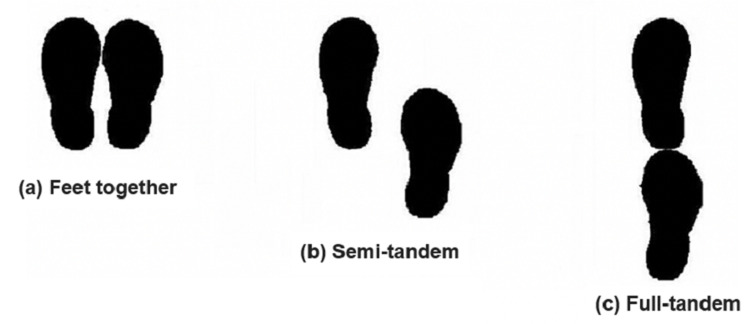
Feet marks of the feet: (a) together feet, (b) semi-tandem, and (c) full-tandem standing

We asked the subject/patient to put his/her feet together and stand for a duration of more than 10 seconds, which was measured using a stopwatch as shown in Figure 1. He/she was allowed to use his/her arms, bend knees, or move their body in order to maintain his/her balance while trying not to move their feet [7,9].

1. Those subjects/patients who were able to stand for a duration of 10 seconds were allotted one point and were thereby promoted for the semi-tandem stand.

2. Those subjects/patients were allotted zero points who were unable to stand for a duration of 10 seconds, and they were then forwarded for the gait speed test.

For the semi-tandem stand, the same procedure was repeated where the heel of one foot was placed by the big toe of the other foot. The patient was allowed to keep either of the foot in the front (whichever was more comfortable for the patient), and the test was then stopped when the patient moved his/her feet or grasped the examiner for support or when the test was completed.

The test was again repeated for a full-tandem stand with the feet directly in front of each other with either foot in the front for a duration of 10 seconds while the examiner was standing behind the subject/patient for the purpose of protection.

1. Those patients who were able to stand for a duration of 10 seconds were allotted two points.

2. Those patients who were able to stand for a duration of 3 seconds to 99.9 seconds were allotted one point.

3. Those patients who were not able to stand or stood for a duration of fewer than 3 seconds were allotted zero points.

All the patients were examined for the speed of gait. To check the gait speed, the patients were asked to walk a zone of premeasured 4 m at a normal pace. Patients were then allowed to use the assistive device if needed by them. Also, the patients were now instructed not to slow their pace down before reaching the endpoint. Using the following system, points were allotted: <4.82 sec (four points); 4.83-6.20 (three points); 6.21-8.70 (two points); >8.71 (one point); and unable to do (zero points) [7,9].

All the patients were taken for the chair stand (rise) test. To check the chair stand test, the patients were asked to stand from the sitting position from the chair (with armrest) with arms folded across the chest without the use of the armrest of the chair for a total of five times as rapid as possible without stopping. The stopwatch was started as soon as the patient stated that he/she was ready and bent forward at his/her hips. The number of stands were counted loudly and stopped at the completion of the fifth stand or if the patient was unable to complete five rises and if the patient’s safety was compromised due to the presence of imbalance [7,9].

The score was awarded according to the following norms:

1. If the subject completed the test in ≤11.9 seconds (four points); 11.20-13.69 seconds (three points); 13.70-26.69 seconds (two points); 16.70-59.99 seconds (one point).

2. If the subject was unable to do the test or unable to complete the test in 60 seconds, then he/she was given zero points.

Gait speed of <0.82 m/s was considered as an indicator of sarcopenia [7,9,10]. A total SPPB score of <7 was considered as an indicator of sarcopenia [7,9,10].

Statistical analysis

All the data were coded and recorded in MS Excel (Microsoft Corporation, Redmond, Washington, USA). Descriptive statistics were elaborated in the form of mean ± standard deviation for continuous variables and frequencies/percentages for categorical variables. Logistic regression analysis was used to obtain the odds ratio (OR). IBM SPSS (Statistical Package for the Social Sciences) statistics v23 (IBM Corp., Armonk, NY) was used for statistical analysis. Statistical significance was set at p-value < 0.05.

**Note: **An unauthorized version of the English MMSE was used by the study team without permission, however this has now been rectified with PAR. The MMSE is a copyrighted instrument and may not be used or reproduced in whole or in part, in any form or language, or by any means without written permission of PAR (www.parinc.com).

## Results

Out of total 70 patients enrolled in this study, 42 were in sarcopenic groups and 28 in nonsarcopenic groups; mean age being 68.61±5.88 years and 69.10±6.31 respectively. In sarcopenic groups 28(66.7%) patients and in non sarcopenic groups 22(78.58%) were male. 18(42.86%) patients were severe frail in sarcopenic group whereas 8(28.57%) patients in nonsarcopenic group. Other base line characteristics along with diagnosis at the time of admission are shown in table 3. Table 3 shows that there was a statistically significant correlation between the sarcopenic and non-sarcopenic groups and hypertension as far as diagnosis is concerned (p = 0.002), and no other baseline characters had any significant correlation with the sarcopenic and non-sarcopenic groups.

**Table 3:** Baseline characteristics DM, Diabetes mellitus; HTN, hypertension; CKD, chronic kidney disease; ACS, acute coronary syndrome; CVE, cerebrovascular episode.

Characteristics	Sarcopenic (n = 42)	Non-sarcopenic (n = 28)	p-value
Age (years)	68.61 ± 5.88	69.10 ± 6.31	0.74, NS
Gender			
Male	28 (66.7%)	22 (78.58%)	0.28, NS
Female	14 (33.3%)	6 (21.42%)	
Frailty Index Score (MFI)	0.52 ± 0.31	0.43 ± 0.28	0.532, NS
Fit	14 (33.33%)	14 (50%)	
Mild	4 (9.52%)	2 (7.14%)	
Moderate	6 (14.29%)	4 (14.29%)	
Severe	18 (42.86%)	8 (28.57%)	
Diagnosis			
Sepsis	14 (33.33%)	12 (42.86%)	0.14, NS
CKD	3 (7.14%)	2 (7.14%)	1.00, NS
DM	5 (11.90%)	4 (14.29%)	0.67, NS
HTN	23 (54.76%)	8 (28.57%)	0.0002, S
DM + HTN	9 (21.43%)	5 (17.86%)	0.59, NS
Cirrhosis	3 (7.14%)	3 (10.71%)	0.32, NS
ACS	9 (21.43%)	6 (21.43%)	1.00, NS
CVE	0 (0%)	1 (3.57%)	0.17, NS

Table 4 shows the association of sarcopenia with outcomes. It shows that there is statistically no significant correlation between the sarcopenic and non-sarcopenic groups and outcomes like mortality, need for ventilation, and length of ICU stay. The p values are >0.05 and are statistically non-significant.

**Table 4:** Association of sarcopenia with outcomes

Characteristics	Sarcopenic (n = 42)	Non-sarcopenic (n = 28)	p-value
Mortality	10 (23.80%)	4 (14.28%)	0.32, NS
Intensive Care Unit stay (in days)	4.30 ± 1.07	3.85 ± 1.23	0.10, NS
Need for ventilation	10 (23.80%)	4 (14.28%)	0.32, NS

Out of 42 patients in sarcopenic groups 10(23.80%) had mortality, length of ICU stays was 4.30±1.07 days and 10(23.80%) required ventilation as shown in table 4. There was no statistically significance between sarcopenic and non sarcopenic group and outcomes like Mortality, need of ventilation and Length of ICU stay.

Table 5 shows the association of sarcopenia, frailty index, and its outcome. This table shows that there is a statistically significant correlation between outcomes like discharge, mortality, and need for ventilation and frailty types in the sarcopenic and non-sarcopenic groups with p = 0.0001. 

**Table 5:** Association of sarcopenia, frailty index, and its outcome

Characteristics	Outcome			p-value
	Discharge	Mortality	Ventilated	
Sarcopenic frailty type				
Non-frail	14 (33.33%)	0 (0%)	0 (0%)	0.001, S
Mildly frail	2 (4.76%)	1 (2.38%)	1 (2.38%)	
Moderately frail	2 (4.76%)	3 (7.14%)	1 (2.38%)	
Severely frail	4 (9.52%)	6 (14.29%)	8 (19.05%)	
Total	22 (52.38%)	10 (23.81%)	10 (23.81%)	
Non-sarcopenic frailty type				
Non-frail	14 (50%)	0 (0%)	0 (0%)	0.0001, S
Mildly frail	2 (7.14%)	0 (0%)	0 (0%)	
Moderately frail	4 (14.29%)	0 (0%)	0 (0%)	
Severely frail	0 (0%)	4 (14.29%)	4 (14.29%)	
Total	20 (71.43%)	4 (14.29%)	4 (14.29%)	

Out of 42 patients in sarcopenic groups, mortality was 1(2.38%) in mild frail type, 3(7.14%) in moderate type and 6(14.29%)in severe frail type. Ventilation were required in 1(2.38%) in mild and moderate frail type whereas 8(19.05%) in severe frail types [p= 0.001], shown in Table 5.

Table 6 shows the association of sarcopenia with H/O major illness. It shows that there is a statistically significant correlation between hypertension and the sarcopenic and non-sarcopenic groups with p = 0.0002.

**Table 6:** Association of sarcopenia with a history of major illness

H/O Major Illness	Sarcopenic (n = 42)	Non-sarcopenic (n = 28)	Total (n = 70)	p-value
Sepsis	14 (33.33%)	12 (42.86%)	26 (37.14%)	0.14, NS
Chronic kidney disease	3 (7.14%)	2 (7.14%)	5 (7.14%)	1.00, NS
Diabetes mellitus	5 (11.90%)	4 (14.29%)	9 (12.86%)	0.67, NS
Hypertension	23 (54.76%)	8 (28.57%)	31 (44.29%)	0.0002, S
Diabetes mellitus + hypertension	9 (21.43%)	5 (17.86%)	14 (20%)	0.59, NS
Cirrhosis	3 (7.14%)	3 (10.71%)	6 (8.57%)	0.32, NS
Acute coronary syndrome	9 (21.43%)	6 (21.43%)	15 (21.43%)	1.00, NS
Cerebrovascular episode	0 (0%)	1 (3.57%)	1 (1.43%)	0.17, NS

Association of Sarcopenia with Chronic illness has been highlighted in table number 6. It showed statistically significant correlation between hypertension and sarcopenic and non-sarcopenic groups P=0.0002.

Table 7 shows a binary logistic regression analysis. The logistic regression analysis shows that there is a statistically significant correlation between mid-upper arm circumference, short physical performance battery, hypertension, diabetes mellitus + hypertension, and cerebrovascular episode with p = 0.0001.

**Table 7:** Logistic regression analysis DM, Diabetes mellitus; HTN, hypertension; ACS, acute coronary syndrome; CVE, cerebrovascular episode; MUAC, mid-upper arm circumference; CC, chest circumference; SPPB, short physical performance battery.

Binary logistic regression	B	S.E.	Wald	Df	p-value	OR
Age	-0.19	620.85	0.11	1	0.738, NS	0.825
Gender	0.5	5519.71	1.16	1	0.280, NS	1.651
Frailty score	0.21	12356.3	1.12	1	0.112, NS	1.621
MUAC (cms)	0.37	1715.35	4.54	1	0.033, S	1.451
CC (cms)	-0.56	1539.82	0.89	1	0.345, NS	0.571
SPPB	34.72	3676.53	46.52	1	0.0001, S	1.20E+15
BMI	0.42	1588.08	0.13	1	0.712, NS	1.523
Length of ICU stay	-1.22	3951.21	10.24	1	0.001, S	0.295
Sarcopenic	-173.52	155974				
Diagnosis						
Sepsis	0.31	1529.21	0.82	1	0.312, NS	0.652
CKD	0.51	1259.6	0.89	1	0.415, NS	0.316
DM	1.29	1566.02	0.91	1	0.319, NS	0.416
HTN	25.65	3579.23	41.52	1	0.0001, S	1.516
DM + HTN	33.63	3921.23	42.61	1	0.0001, S	1.295
Cirrhosis	-3.22	2965.31	31.52	1	0.796, NS	1.329
ACS	-5.21	1956.32	21.53	1	0.516, NS	1.315
CVE	0.31	1521.36	3.21	1	0.033, S	1.236

The logistic regression analysis shows that there was a statistically significant correlation between MUAC, SPPB, HTN, DM+HTN and CVE with P=0.0001 as shown in table 7. 

## Discussion

As medical science is advancing fast, the population pyramid has undergone very significant changes in terms of increased life expectancy in the elderly, hence more frequent sarcopenia and frailty. These conditions must be identified and treated promptly to minimize the health consequences for the critically affected elderly. The sarcopenia was present in 60% of our study population and settings, which differs from the published results probably due to the methods used for evaluating this condition as most studies have been carried out in the community or in nursing homes [2,4].

In our study, the sarcopenic patients were frailer. In a study by Joyce et al., it was observed that sarcopenia was highly prevalent in the ICU population (68%) [15]. Few studies have reported only 10% of cases in acute care hospitals; however, it may be underreporting of cases as gait speed or handgrip strength were not able to be measured in 22.3%, thus lacking at least one of the EWGSOP criteria for diagnosis [16]. In our study, there is a significant correlation between the sarcopenic, non-sarcopenic groups, and hypertension (p = 0.0002), whereas in the other study [15], a significant correlation was between the sarcopenic, non-sarcopenic groups, and age. As far as gender is considered, sarcopenia is higher in women. Few studies had shown an estimated prevalence of 15.3% in men and 20.5% in women in a tertiary-care hospital [16,17].

In the SABE (health, well-being, and aging) study carried out in Colombia, the condition was also higher in women (12.6% vs 9.8%), contrary to what we found in our study where men were the most affected (66.7% vs 33.3%) [18]. The gender difference, in our study, was not statistically significant. In our study while comparing outcomes among the sarcopenic and non-sarcopenic groups, there was not any statistically significant correlation; 42.86% of patients were severely frail in the sarcopenic group, whereas 28.57% of patients were severely frail in the non-sarcopenic group in our study, and there was a significant correlation between the sarcopenic and non-sarcopenic groups with frailty type with their outcome.

We could not find any study after extensive research correlating sarcopenia with muscle fat infiltration (MFI) and outcomes. Sarcopenia and frailty are known to be closely related as geriatric syndromes and may coexist. In our patients, these two entities were found simultaneously, whereas, in a study performed in the Netherlands [19], the intra-individual correlation between sarcopenia and frailty was low. A possible explanation may be the difference in pathophysiology, since sarcopenia is based on musculoskeletal alterations, while frailty has a complex multifactorial cause including emotional and cognitive elements, according to the assessment tool (MFI used in this study). More studies with a larger population are needed to determine this relationship.

**Figure 2:**
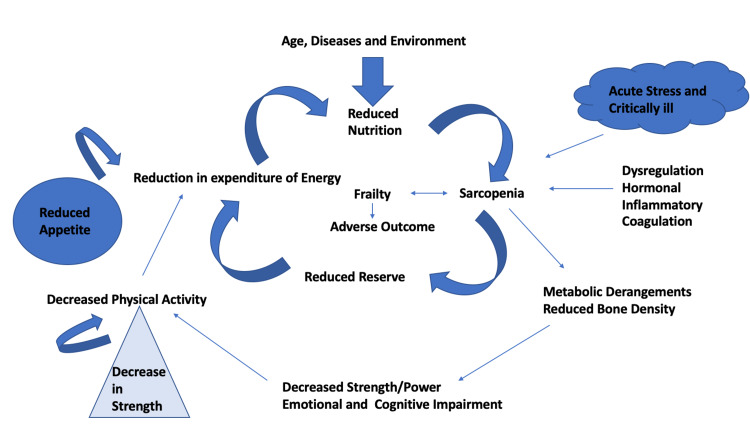
Overview of the vicious cycle of frailty VO2 max, Maximal oxygen consumption.

In a study by Mohanty et al. [16], the length of ICU stays increased from 1.66 to 3.75 days (p < 0.001) as the FI increased, whereas in our study also, the mean length of ICU stay was 4.07 days in the sarcopenic group and 3.28 days in the non-sarcopenic group. There was a statistically significant correlation between the length of ICU stay and the sarcopenic and non-sarcopenic groups in the study by Mohanty et al. [16], whereas, in our study, no such correlation was associated. In a study by Zhang et al. [17], age was found to be an important confounding factor, whereas, in our study, we did not have age as a confounding factor. Also, in addition, there were a number of important confounding factors, such as chronic obstructive pulmonary disease (COPD), obesity, and cardiac failure, which would have influenced sarcopenia’s impact on mortality, whereas, in our study, no such confounding factors were observed. There was a statistical significance between outcomes like discharge, mortality, and need for ventilation and frailty types in the sarcopenic and non-sarcopenic groups with p = 0.0001. In our study, there were definite adverse outcomes like mortality in ICU patients, need for a ventilator, and more duration of ICU stay in patients of sarcopenia, and it was an additive effect as frailty index increased. The pathophysiology of critically ill patients becoming sarcopenic and frail is explained in Figure 2.

The logistic regression analysis showed that there was a statistically significant correlation between id-upper arm circumference, SPPB, hypertension, diabetes mellitus + hypertension, and cerebrovascular episode with p = 0.0001 in the sarcopenic patients with different levels of the frailty index. However, more studies are required to justify this as data is lacking in the literature.

Limitations

The main limitation of this study is the small sample size because of the short-term study. We could not follow up with the patients. In our study, we could not assess sarcopenia by muscle mass estimation of psoas muscle by CT scan due to financial constraints, which is the gold standard method for estimating the muscle mass.

## Conclusions

Our study found that critically ill patients with sarcopenia had an increased risk of mortality compared to those without sarcopenia. Timely routine assessment for sarcopenia upon ICU admission may provide an important prognostic factor in patient survival. Offering corresponding interventions may help medical staff achieve good patient outcomes. A more extensive and larger sample-sized study should be conducted before any conclusion.
