# Correction: Correlation of Sarcopenia With Modified Frailty Index as a Predictor of Outcome in Critically Ill Elderly Patients: A Cross-Sectional Study

**DOI:** 10.7759/cureus.c385

**Published:** 2025-12-19

**Authors:** Shreerang Bhurchandi, Sunil Kumar, Sachin Agrawal, Sourya Acharya, Shraddha Jain, Dhruv Talwar, Sunayana Lomte

**Affiliations:** 1 Department of Medicine, Jawaharlal Nehru Medical College, Datta Meghe Institute of Medical Sciences, Wardha, IND; 2 Department of Otorhinolaryngology, Jawaharlal Nehru Medical College, Datta Meghe Institute of Medical Sciences, Wardha, IND

This article has been corrected to include the following statement at the end of the Materials and Methods section:

**Note: **An unauthorized version of the English MMSE was used by the study team without permission, however this has now been rectified with PAR. The MMSE is a copyrighted instrument and may not be used or reproduced in whole or in part, in any form or language, or by any means without written permission of PAR (www.parinc.com).

